# Dietary chia (*Salvia hispanica* L.) improves the nutritional quality of broiler meat

**DOI:** 10.5713/ajas.19.0608

**Published:** 2019-10-22

**Authors:** Nicole Batelli de Souza Nardelli Mendonça, Sérgio Turra Sobrane Filho, David Henrique de Oliveira, Eduardo Machado Costa Lima, Priscila Vieira e Rosa, Peter Bitencourt Faria, Luciana de Paula Naves, Paulo Borges Rodrigues

**Affiliations:** 1 Department of Animal Science, Federal University of Lavras, Lavras, MG 37200-000, Brazil; 2 Department of Veterinary Medicine, Federal University of Lavras, Lavras, MG 37200-000, Brazil

**Keywords:** Chia Oil, Chia Seed, Fatty Acid Profile, Food Enrichment, Omega-3, ω-3

## Abstract

**Objective:**

The current study was conducted to evaluate the quality and profile of fatty acid in the breast and thigh, and the performance of broilers fed diets containing seed or oil of chia (*Salvia hispanica L*.) as a replacement for soybean, in the rearing period from 29 to 42 days of age.

**Methods:**

On the 29th day of age, 120 broilers were distributed in four treatments evaluated in five replicates of six birds. The grain or oil of soybean was respectively replaced on a weight-to-weight basis in the formulation by the seed or oil of chia, constituting the experimental diets. The roasted whole soybean and chia seed were included in the feed at 16.4%, whereas the soybean and chia oils were included at 2.5%.

**Results:**

The dietary chia oil increased the lipid peroxidation in the thigh meat, and the dietary chia seed increased the cooking loss of the thigh. However, for the other physicochemical parameters evaluated and for the proximate composition of the breast and thigh, in general, the inclusion of chia seed or oil in the diet provided similar or better results than those observed when the diets contained soybean oil or roasted whole soybean. With regard to the fatty acid profile and associated parameters, dietary chia increased the concentrations of α-linolenic, eicosapentaenoic, and docosahexaenoic acids and reduced the ∑*ω*-*6*:∑*ω*-*3* ratio and the atherogenicity and thrombogenicity indices of the broiler meat. However, the dietary chia seed worsened the feed conversion ratio.

**Conclusion:**

Diet containing 2.5% chia oil supplied to broilers during the period from 29 to 42 days of age improves the feed conversion ratio, increases the deposition of the *ω-3* fatty acids in the breast and thigh, in addition to reducing the ∑*ω-6*:∑*ω-3* ratio and the atherogenicity and thrombogenicity indices, thereby resulting in meat with higher nutritional quality.

## INTRODUCTION

High omega-6:omega-3 (*ω-6*:*ω-3*) ratios in the human diet are associated with a higher risk of obesity and cardiovascular diseases because the imbalanced consumption of fatty acids contributes to atherosclerosis, the main cause of heart attacks and strokes [1]. According to global health agencies, the recommendation of *ω-6*:*ω-3* daily intake converges to ratios between 4:1 and 5:1 [2]. However, especially in the diets of western industrialized countries, the *ω-6*:*ω-3* daily intake can reach ratios higher than 20:1 [1].

The use of nutritional strategies to improve the quality and chemical composition of animal products used in the human diet represents an important link between animal production, food technology, and human nutrition. Broiler meat is more affordable than other meats on the market, being a source of animal protein worldwide used in the human diet. In addition, studies have shown that broiler meat can be enriched with *ω-3* fatty acids by altering the lipid composition of the diets fed to animals [3–5]. Therefore, providing diets rich in *ω-3* for broilers with the goal of enriching their meat with *ω-3* fatty acids is an alternative for increasing the dietary intake of *ω-3* by humans.

However, the increase in the degree of polyunsaturated fatty acid (PUFA) deposition in the meat may increase its susceptibility to lipid peroxidation [4,5] reducing their quality, which can trigger undesirable changes in taste, color, and texture, in addition to a loss in nutritional value [6]. The inclusion of antioxidants in the diets can reduce lipid peroxidation in the meat [7]; however, the use of industrial antioxidants can represent an additional cost when feeding broilers, possibly resulting in an increase in the meat price paid by consumers.

In this sense, chia ( *Salvia hispanica L.*) has the potential to enrich the broiler meat with fatty acids beneficial to human health without compromising the meat quality because this plant source has high concentration of *ω-3* fatty acids and has compounds that exhibit high antioxidant activity [8]. Although some studies have examined the use of chia seed in broiler diet [9,10], chia oil as a dietary source of *ω-3* has not been investigated yet, nor has the supply period of dietary chia been restricted to the last 14 days of rearing. Verify if there is meat enrichment with *ω-3* when broilers receive diet supplemented with chia only in the last two weeks of rearing is important to avoid unnecessary costs with the supply of dietary chia during the total rearing period of the broilers. Therefore, the present study was conducted to evaluate the quality and profile of fatty acid in the breast and thigh, and the performance of broilers fed diets containing seed or oil chia as a replacement for soybean, in the rearing period from 29 to 42 days of age.

## MATERIALS AND METHODS

### Ethics statement

The experiment was conducted in the Poultry Sector of the Department of Animal Science of the Federal University of Lavras (UFLA). All experimental procedures were approved by the UFLA Ethics Committee on Animal Use, under protocol no. 074/16.

### Animals and experimental procedures

A total of 150 one-day-old male Cobb500 broilers were acquired from a commercial hatchery and were reared in a conventional broiler house until 28 days of age, comprising a single batch. During this period, the broilers received basal diets that were formulated to meet their specific nutritional needs according to their age range [11] (Table 1). On the 29th day of age, the broilers were weighed individually and distributed according to weight range for homogeneity. Subsequently, 120 broilers were transferred to a performance-testing broiler house containing boxes measuring 2.0×1.5 meters. Each box (experimental unit) had its floor covered with wood shavings and was equipped with a pendular drinker and feeder.

The experimental design was completely randomized, and diets containing soybean or chia used in the form of grain/seed or oil were evaluated. Therefore, the 120 broilers were distributed into four treatments and evaluated in five replicates (box) consisting of six birds each. The lighting program adopted was continuous light, with 24 hours of light throughout the experimental period. The minimum temperature recorded in the broiler house was 19.7°C±0.5°C, and the maximum temperature was 31.8°C±1.3°C.

### Experimental diets and growth performance

The experimental diets (Table 1), which were based on corn and soybean meal, were formulated according to the recommendations of Rostagno et al [11] and were supplied *ad libitum* to the animals during the period when they were 29 to 42 days of age. Initially, the control diet containing corn, soybean meal and soybean oil (2.5%) was formulated. Subsequently, an isonutrient diet without soybean oil and containing roasted whole soybean (16.4%) was formulated. After, the soybean grain/oil was replaced on a weight-to-weight basis in the formulation by the chia seed/oil, constituting the other experimental diets. Thus, the roasted whole soybean and chia seeds were included in the feed at 16.4%, whereas the soybean and chia oils were included in the feed at 2.5%. The chia seed was added whole to the feed because according to Nitrayová et al [13] the chia seed not need to be ground to release its nutrients. The chia seeds were purchased from a local market in São Paulo, SP, Brazil; the chia oil in Rio de Janeiro, RJ, Brazil; and roasted whole soybean in Três Corações, MG, Brazil. The other ingredients were purchase from a local market in Lavras, MG, Brazil. The chemical composition and gross energy of the evaluated feedstuffs is related in the Table 2.

The fatty acid profiles of the foods tested and of the ex perimental diets (Tables 3, 4, respectively) were determined by gas chromatography in a Shimadzu CG 2010 chromatograph (Agilent Technologies Inc., Palo Alto, CA, USA), using the lipid extraction method described by Folch et al [14] and the esterification method proposed by Hartman and Lago [15].

The broilers, feeds and leftovers were weighed on days 29 and 42 to calculate weight gain, feed intake, and feed conversion ratio. No mortality occurred during the experimental period.

### Slaughter and procedures for collection and storage of the broiler breast and thigh

At 42 days of age, after 12-hours of fasting, three broilers per experimental unit were randomly sampled, for a total of 15 broilers per treatment. The selected broilers were slaughtered by cervical dislocation, followed by bleeding, scalding, plucking, and evisceration. Subsequently, the breast (*pectoralis major* muscle) and thighs (*fibularis longus*, *tibial cranial*, *flexor digitorum longus*, *extensor digitorum longus*, and *gastrocnemius* muscles) were collected, individually packed in labeled plastic bags, and cooled at 5°C for 24 hours.

After this cooling period, the breast and thigh samples were immediately analyzed for pH 24 hours *postmortem* (pH_24h_), color, cooking loss (CL), and shear force (SF). In addition, aliquots of the cooled breast and thigh samples were frozen at −18°C for 10 days, at which point the thiobarbituric acid-reactive substances (TBARS), proximate composition, and fatty acid profile were analyzed.

### Physicochemical analyses of meat

The pH_24h_ readings were taken using a digital pH meter (Hanna Instruments, Model HI 99163, São Paulo, Brazil). Color was determined on the upper surface of the samples after exposure to the ambient air for 30 minutes. The lightness (L*), red intensity (a*), and yellow intensity (b*) parameters were determined using a colorimeter (Konica Minolta, Chroma Meter-200b, Tokyo, Japan) operating in the CIELAB system with illuminant D65, calibrated to standard white tile. The saturation index (C*) and the hue angle (h*) were calculated by the equations C* = (a*2 + b*2) 1/2 and h* = tan−1 (b*/a*) [16].

After the color analysis, aliquots of the samples were weigh ed, wrapped in aluminum foil, and cooked in an electric hotplate preheated to 150°C±5°C. After reaching a temperature of 35°C, the samples were flipped so that the heating was homogeneous and allowed to continue cooking until the internal temperature reached 72°C±2°C. After being cooked, the samples were refrigerated at 5°C and then weighed, with the CL being determined by the difference between the weight before and after cooking [16].

To evaluate the SF, the cooked samples used in the CL analysis were cut into pieces smaller of 2.0×1.0×1.0 cm and then they were sectioned perpendicular to the muscle fibers using a texture analyzer (Extralab, Model TA.XT Plus, São Paulo, Brazil). The SF results were expressed in kilogram-force (kgf).

### Lipid stability and proximate composition of the meat

After storage at −18°C for 10 days, the breast and thigh samples were thawed under refrigeration at 5°C for 24 hours, after which the TBARS content was measured according to the methodology proposed by Tarladgis et al [17]. The malondialdehyde (MAD) concentration was determined from a standard calibration curve with 1,1,3,3-tetraethoxypropane, and the results were expressed in milligrams of MAD/kg of sample.

The proximate composition (moisture, protein, lipids, and mineral matter) of the breast and thigh was determined in skinless samples, ground, and homogenized using the FoodScan Meat Analyzer (FOSS, Hillerod, Denmark), according to AOAC official method of number 2007.04 [18].

### Determination of the meat fatty acid profile

The profile of fatty acids in broiler breast and thigh was determined using the lipid extraction method described by Folch et al [14] and the esterification method proposed by Hartman and Lago [15]. Fatty acids were identified in a Shimadzu GC 2010 gas chromatograph (Agilent Technologies Inc., USA) equipped with a flame ionization detector, a split injector at 1:50 split ratio, and a Supelco SPTM-2560 capillary column (100 m×0.25 mm×0.20 m; Supelco Inc., Bellefonte, PA, USA).

The chromatographic conditions were an initial column temperature of 140°C/5 min, with an increase of 4°C/min up to 240°C and remaining at this temperature for 30 minutes for a total runtime of 60 minutes. The injector and detector temperatures were 260°C. The carrier gas used was helium. Fatty acids were identified by comparison with retention times characteristic of chromatographic standards (Supelco 37 standard FAME Mix, Supelco Inc., USA) and were expressed as percentages of the total fatty acids identified. The total saturated fatty acids (SFA), monounsaturated fatty acids (MUFA), PUFA, total *ω-3* fatty acids (∑*ω-3*), and total *ω-6* fatty acids (∑*ω-6*) were calculated by the sum of the individual contributions of the acids.

The activity indexes of the Δ9-desaturase and elongase enzymes were estimated according to Malau-Aduli et al [19]. The atherogenicity and thrombogenicity indexes were calculated according to Ulbricht and Southgate [20] using the following equations: the atherogenicity index = (4 [C14:0]+ C16:0)/(∑MUFA+∑PUFA), and the thrombogenicity index = (C14:0+C16:0+C18:0)/([0.5×∑MUFA]+[0.5×∑ω-6]+ [3×∑ω-3]+[∑ω-3/ω-6]).

### Statistical analyses

The data were analyzed using the Bartlett’s and Shapiro-Wilk tests, at the p<0.05 statistical level, to test for the assumptions of the analysis of variance (ANOVA) (homogeneity of variances and normality of errors). If one of the assumptions was not met, the data were logarithm-transformed for subsequent statistical analysis. If both assumptions were met, the data were analyzed by ANOVA, and the means were compared by the Scott Knott test at 5% probability, using the statistical program R (version 3.2.5, 2017, R Core Team, Vienna, Austria).

## RESULTS

### Physicochemical parameters and lipid peroxidation of meat

The results of pH24h, color (L*, a*, b*, C*, and h*), CL, SF, and lipid peroxidation of the broiler breast and thigh are reported in Table 5. The breast of the broilers fed diet containing chia seed had lower pH_24h_ (p<0.05), whereas the highest pH_24h_ (p<0.05) was found in the breast of broilers fed diets containing soybean oil. In the thigh, the lowest pH_24h_ (p<0.05) was observed for broilers fed diet with soybean oil, with no differences observed in the pH_24h_ of the thigh among the birds receiving any of the other experimental diets.

The experimental diets did not influence (p >0.05) the parameters L* and h* in the breast meat of broilers. However, the inclusion of roasted whole soybean or chia seeds in the diet resulted in a higher (p<0.05) a* value in the breast. For the parameters b* and C*, the lowest values (p<0.05) were observed in the breast of broilers fed diet containing soybean oil, with no differences observed among the broilers fed the other diets evaluated.

The experimental diets did not influence (p >0.05) the L*, a*, and C* parameters in the thigh. However, lower b* (p< 0.05) was observed in the thighs of birds that received a diet containing soybean oil. Regarding the parameter h*, the thigh of the birds fed diet containing the chia seed had the highest value (p<0.05).

Breast CL did not change (p >0.05) according to the diet provided to the broilers. However, the inclusion of chia seed in the diet increased (p<0.05) the thigh CL. The SF in the breast and thigh meat was not affected (p>0.05) by the diet provided to the broilers.

The TBARS content in the broiler breast was not affected (p>0.05) by the experimental diets. However, higher lipid peroxidation (p<0.05) was observed in the thighs of broilers fed diet containing chia oil.

### Proximate composition of the meat

The moisture and lipid levels in the breast and thigh of the broilers were not (p>0.05) influenced by the diet provided to the birds (Table 6). Although the protein content in the thigh was not altered (p>0.05) by the different diets evaluated, the use of roasted whole soybean or chia seeds in the diet increased (p<0.05) the protein content in the breast. The diet containing soybean oil increased (p<0.05) the deposition of minerals in the breast, whereas the diet containing chia seed increased the mineral matter content in the thigh (p<0.05).

### Meat fatty acid profile

The fatty acid profile of the breast is shown in Table 7. The breast of broilers fed diet containing soybean oil exhibited the highest (p<0.05) levels of C18:0, C18:1*n-9 trans*, C20:3*n-6*, C24:0, and total SFA; the lowest (p<0.05) PUFA/SFA ratio; and the highest (p<0.05) thrombogenicity index. In turn, the inclusion of chia seed in the diet resulted in the lowest (p< 0.05) deposition of C6:0, C17:0, and C18:2*n-6cis*; the highest (p<0.05) deposition of C16:1, C20:5*n-3*, and C22:2; and the highest (p<0.05) Δ9-desaturase^C16^ enzyme activity in the breast of the broilers.

The highest (p <0.05) C18:3*n-3* enrichment in the breast occurred when the broilers were fed the diet containing chia oil, followed by the diet containing the chia seed. An increase (p<0.05) in the total PUFA content and a decrease (p<0.05) in the C20:1*n-9* content was observed in the breast only when the chia oil was included in the diet. However, both the dietary chia oil and seed reduced (p<0.05) the concentrations of C18:3*n-6*, C20:2, C20:4*n-6*, and ∑-*6*; decreased (p<0.05) the ∑*ω-6*/∑*ω-3* ratio; and increased (p<0.05) the deposition of C20:3*n-3*, C22:6*n-3*, and ∑*ω-3* in the broiler breast. In turn, lower (p<0.05) C16:0 concentration and lower (p<0.05) atherogenicity index were observed in the breast of the broilers that received the diet containing chia oil or roasted whole soybean.

The fatty acid profile of the broiler thigh is shown in Table 8. The thigh of the broilers fed diet containing roasted whole soybean had the lowest (p<0.05) C17:0 content. The inclusion of chia seed oil in the diet increased (p<0.05) the levels of C20:0 and total PUFA, increased (p<0.05) the PUFA:SFA ratio, and decreased (p<0.05) the atherogenicity index in the broiler thighs. However, both the dietary chia oil and seed increased (p<0.05) the concentrations of C18:3*n-3*, C20:5*n-3*, C22:2, and ∑*ω-3* and decreased (p<0.05) the concentrations of C18:1*n-9cis*, C18:3*n-6*, total MUFA, the ∑*ω-6*/∑*ω-3* ratio, the Δ9-desaturase^C18^ enzyme activity, and the thrombogenicity index in the thighs of the broilers.

The highest (p <0.05) enrichment in C20:3*n-3* and C22:6*n-3* in the thigh occurred when the broilers were fed diet containing chia oil, followed by the diet containing chia seed. The lowest (p<0.05) levels of C18:2*n-6cis* and ∑*ω-6* were observed in the thighs of the birds that received the diet with chia seed, whereas the highest amounts of these fatty acids were measured in the thigh of broilers fed the diet that contained soybean oil or roasted whole soybean.

### Growth performance

The performance results are reported in Table 9. The broilers fed diets containing soybean oil or chia seed exhibited higher (p<0.05) feed intake. The inclusion of soybean oil or chia oil in the diet resulted in a higher (p<0.05) weight gain of the broilers. However, the best (p<0.05) feed conversion ratio was observed when the broilers received the diet containing chia oil or roasted whole soybean.

## DISCUSSION

The pH_24h_ values of the breast measured in the present study ranged from 5.73 to 5.93, similar to the range of 5.64 to 5.93 reported in the study by Betti et al [3], who evaluated the potential of dietary flax for *ω-3* enrichment of broiler breasts. Already the pH_24h_ values measured in the thigh (6.34 to 6.46) were higher than the mean of 6.02 reported for male Cobb broiler fed basal diet [21].

In general, the higher pH _24h_ values obtained in the thigh compared to the breast can be explained by the type of muscle fibers predominant in each cut. In the breast, white muscle fibers, which have a higher *postmortem* pH decline rate that results in lower pH_24h_ values, are predominant [22]. In the thigh, red muscle fibers predominate, and these have a lower *postmortem* pH decline rate due to the lower capacity to store glycogen and to metabolize it via the anaerobic pathway [6].

The present study demonstrates that the diet composition has a greater effect on the pH_24h_ of the breast because the diets containing chia oil, roasted whole soybean, and chia seed influenced the pH_24h_ of the breast, although they did not alter the pH_24h_ of the thigh. Because a close relationship exists between the pH_24h_ and the rates of storage and degradation of glycogen in lactic acid in the *postmortem* period, the results suggest that these diets may have altered the glycogen concentration and/or its anaerobic degradation rate, especially in the breast, which is a cut composed predominantly of muscle fibers highly specialized in storing and metabolizing the glycogen in lactic acid [22].

The L* parameter is related to the brightness on the surface of the meat cut. The L* value has been negatively correlated with the pH_24h_ value of the meat with the explanation that a higher pH decline rate may cause a greater denaturation degree of the myoglobin pigments [23] and greater liquid exudation, promoting greater light dispersion on the surface of the meat [24]. However, in the present study, such correlation between pH_24h_ and L* was not observed because the changes in pH_24h_ values did not affect the L* parameter in both cut evaluated.

The greater the myoglobin content in the muscle, the higher the a* [23]. In the present study, the a* value in the thigh, on average, was approximately 6.5 times higher than in the breast, which may be explained by the fact that the thigh is a cut that naturally has a greater amount of red muscle fibers rich in myoglobin [22]. The inclusion of roasted whole soybean or chia seeds in the diet resulted in higher a* in the broiler breast, suggesting that part of the chemical compounds present in these foods may have been metabolized and used for the synthesis of myoglobin. On the other hand, as the thigh naturally has a higher red content, the results suggest that the contribution of these dietary ingredients as precursors in myoglobin biosynthesis was not large enough to change the a* value in the thigh.

The effects of the experimental diets on the b* parameter were the same in the breast and thighs of the birds. The higher b* value in the breast and in the thigh of the broilers that received the diet containing chia oil, roasted whole soybean, or chia seed are likely attributable to these ingredients presenting a more diverse pigment profile when compared to the soybean oil [25,26].

The C* parameter indicates the degree of color saturation of the meat. Using Pearson’s correlation analysis, Bridi et al [27] reported a negative correlation between the pH_24h_ value and the C* value. In general, in the present study, the effects of the experimental diets on pH_24h_ were not reflected in the C* value, except for the breast of the broilers fed diet containing soybean oil, which resulted concomitantly in the highest pH_24h_ value and in the lowest C*. In turn, the h* parameter indicates the predominant hue of the meat. Thus, both the breast and thigh meat of the broilers can be considered yellow because this color is defined in the color scale of the CieL*a*b* system for h* values between 70 and 100 degrees. However, although in the CieL*a*b* system the h* values for the breast and thigh were in the same quadrant, the mean h* measured in the breast was 87.31°, which is closer to light yellow, whereas the mean h* measured in the thigh was 73.8°, which was closer to the orange quadrant.

In the present study, the mean CL in the breast was 27.96%, which is similar to that reported by Betti et al [3], who also studied *ω-3* enrichment in broiler meat. The CL values of the thigh in the present study ranged from 24.57% to 28.94%, which is lower than the mean value of 30.6% reported by Wan et al [7]. However, in the study by Wan et al [7], the mean pH measured in the thighs of the broilers was lower (6.08), which may explain the differences between the two studies.

In the thigh, a higher CL was observed when the broilers received a diet containing chia seeds. Similarly, the dietary chia seed also increased the CL of rabbit meat [28], whose muscle fiber composition is similar to that of a broiler thigh. However, further studies are needed to understand the mechanisms through which chia seed increases the CL in broiler thighs.

Hugo et al [4] and Saleh et al [5] observed increased TBARS values in the breast and thigh of broilers fed diets containing fish oil, demonstrating that the oxidative stability of the lipids present in the meat decreases as the degree of lipid unsaturation increases. In the present study, although the inclusion of chia oil in broiler diets increased the deposition of total PUFA in the breasts, no change occurred in the TBARS content in this cut. According to Marineli et al [8], both the chia oil and the seed contain several phenolic compounds with antioxidant activity, especially myricetin, quercetin, kaempferol, chlorogenic acid, and the 3,4-dihydroxyphenylethanol-elenolic acid dialdehyde. Therefore, the maintenance of lipid stability observed in the breast in the present study can be explained by the presence of antioxidant compounds in the chia.

Contrary to what was observed in the breast, the inclusion of chia oil in the diet of broilers increased both the total PUFA deposition and the MDA concentration in the thigh, indicating that, in this cut, the action of the antioxidant compounds present in the chia oil was not high enough to avoid an increase in lipid peroxidation. This result can be explained by the fact that the lipid content in the thigh, on average, was 2.5 times higher than in the breast. The higher fat content of the thigh compared to the breast is due to the higher concentration of red muscle fibers in the thigh, which can metabolize triacylglycerols for energy production [6].

According to Rymer and Givens [29], broiler meat with higher linolenic acid content is more susceptible to oxidation. In the present study, diets containing chia oil or seed resulted in higher deposition of C18:3*n-3* in the broiler thigh. However, higher MDA content was observed only in the thighs of the birds that received the diet containing chia oil, indicating that chia oil had lower antioxidant activity than chia seed. Marineli et al [8] showed that chia oil has a lower amount of antioxidant compounds and a higher rate of autoxidation of these compounds than the chia seed, which may explain the results observed in the present study.

The use of roasted whole soybean and chia seeds in the diet resulted in higher protein deposition in the breast compared to the dietary use of soybean and chia oils. In diets containing roasted whole soybean and chia seed, the inclusion level of the industrial amino-acid *L*-valine was twice that used in the formulation of diets containing soybean oil and chia, which may have contributed to the results for protein deposition in the breast of the broilers. The valine, together with leucine and isoleucine, plays an important role in protein synthesis. According to Wu [30], valine is directly related to glutamine synthesis and branched chain amino acid (BCAA) balance. According to Watford and Wu [31], a directly proportional relationship exists between intramuscular glutamine concentrations and muscle protein synthesis in broilers. In addition, simultaneous supplementation of all BCAAs, including valine [30], is necessary for leucine to achieve its full potential in muscle growth. Therefore, diets containing roasted whole soybean or chia seed may have favored glutamine biosynthesis and improved the BCAA balance, allowing greater protein deposition in the broiler breast.

The fatty acid composition of the breast and thigh of broilers was influenced by the fatty acid profile of the diet, as previously reported in the scientific literature [3–5]. When compared to diets containing soybean oil, broiler diets formulated with chia oil showed α-linolenic acid (C18:3*n-3*) enrichment in the broiler breast and thigh of approximately 336% and 295%, respectively. In contrast, the diet containing chia seed, compared to the diet containing roasted whole soybean, resulted in an increase in C18:3*n-3* of 274% in the breast and 244% in the thigh of the broilers. These results may be explained by the fact that α-linolenic acid is the major fatty acid in chia oil and seeds [8].

The linoleic acid (C18:2 *n-6cis*) content in the chia was lower than that of the soybean. For this reason, the use of chia seed or oil in the diets decreased the C18:2*n-6cis* content in the broiler breasts and thighs. The inclusion of chia oil in the diet, compared to soybean oil, resulted in a 13.1% reduction in C18:2*n-6cis*, both in the breast and in the thigh of the broilers. In contrast, the use of chia seed in the diet, compared to the dietary supply of roasted whole soybean, reduced the C18:2*n-6cis* by 23.1% in the breast and 19.0% in the thigh.

The animal organism is able to convert linoleic and α-linolenic acids into more bioactive components, such as linoleic acid, which is a precursor of arachidonic acid (ARA, C20: 4*n-6*), whereas eicosapentaenoic acid (EPA, C20:5*n-3*) and docosahexaenoic acid (DHA, C22:6*n-3*) are synthesized from α-linolenic acid. However, linoleic and α-linolenic acids compete for the same enzymatic system, especially Δ-6 and Δ-5 desaturase enzymes, which usually exhibit higher affinity for *ω-3* fatty acids [32]. In the breast of broilers fed diet containing chia oil or seed, the results suggest a predominant activity of desaturases in the metabolic pathway of *n-3* fatty acids because of the decrease in the ARA concentration and concomitant increase in the EPA and DHA concentrations. In the thigh, dietary chia also increased the EPA and DHA levels, although it did not alter the ARA concentration, demonstrating that the metabolism of fatty acids is not always identical when comparing cuts with different muscle fiber profiles.

Compared to the diet containing soybean oil, the diet containing chia oil increased the EPA content in the breast by approximately 226%, the EPA content in the thigh by 474%, the DHA content in the breast by 28.8%, and the DHA content in the thigh by 89.4%. Compared to the diet containing roasted whole soybean, the diet containing chia seed increased the EPA content in the breast by approximately 342%, the EPA content in the thigh by 379%, the DHA content in the breast by 55%, and the DHA content in the thigh by 50.8%. Some studies have shown that the concentration of α-linolenic acid, EPA and DHA in the meat of broilers may be increased with the use of chia seed in the diets [9,10]. However, in the cited studies, chia oil as a dietary source of *n-3* was not investigated, and the period of dietary chia supply was longer than the 14-day period evaluated in the present study.

Human food, enriched with *ω-3* PUFA, has gained increasing attention from the consumer market due to the increasingly widespread knowledge of the beneficial effects of these compounds in the promotion of cardiovascular health and even in the prevention and treatment of inflammatory and autoimmune diseases and cancer [33].

Human diets with *ω-6*:*ω-3* ratio close to 4:1 help prevent the development of obesity and inflammatory and cardiovascular diseases [2]. However, human diets in industrialized countries have a high *ω-6*:*ω-3* ratio, reaching values higher than 20:1 [1]. In this sense, the results of the present study are relevant because the use of chia (seed or oil) in the diet of broilers in the period from 29 to 42 days of age promoted the enrichment of the breast and thigh with *ω-3* fatty acids, in addition to having reduced the total concentration of *ω-6* fatty acids in these cuts. Compared to the diet containing soybean oil, the diet containing chia oil reduced the ∑*ω-6*:∑*ω-3* ratio in the breast and thigh by approximately 78.54% and 77.27%, respectively. Compared to the diet containing roasted soybean, the diet containing chia seed reduced the ∑*ω-6*:∑*ω-3* ratio in the breast and thigh by approximately 77.28% and 74.41%, respectively.

The dietary supply of chia seed oil for broilers increased the ∑PUFA:∑SFA ratio in both the breast and thigh meat, which is noteworthy because the consumption of SFA can increase the plasma low-density lipoprotein content, which is a risk factor for cardiovascular diseases [20]. In addition, the lower atherogenicity indices were measured in the breast of birds fed a diet containing chia oil or roasted whole soybean and in the thigh of the broilers that received the diet containing chia oil. Dietary chia, in the form of seed or oil, reduced the thrombogenicity index in the thigh and breast of broilers. The lower the atherogenicity and thrombogenicity indices, the healthier the food can be considered, as a reduction in these indices is associated with a lower risk of atherosclerosis and other cardiovascular diseases related to the lipid composition of the diet [20]. According to Doğan et al [34], although modern broiler lines exhibit fast growth and better performance, they may exhibit changes in meat quality. Therefore, considering the results observed in the present study, meat from broilers fed diet containing chia can be considered healthier than meat from broilers that receive dietary soybean oil.

However, the broilers performance was altered by experimental diets. Although the broilers that received dietary chia seed consumed more feed, they did not exhibit greater weight gain reflecting on worse feed conversion ratio. In aqueous medium a mucilage layer is formed surrounding the chia seed [8], which creates a physical barrier that can reduce the efficiency of digestive enzymes [35] justifying, therefore, the results observed in the current study. On the other hand, the feed conversion rate of the broilers fed diet containing chia oil was better, suggesting that the utilization efficiency of dietary chia oil by broilers was higher than the utilization of dietary chia seed.

## CONCLUSION

The supply of the diet containing 2.5% chia oil for broilers during the period from 29 to 42 days of age enriches the breast and thigh meat with *ω-3* and increases the levels of α-linolenic, eicosapentaenoic and docosahexaenoic acids. In addition, this diet improves the feed conversion ratio and reduces the ∑*ω-6*:∑*ω-3* ratio and the atherogenicity and thrombogenicity indices of the broiler meat, providing improvements in the nutritional quality of the meat, with consequent health benefits for its consumers. However, future studies to evaluate the ideal inclusion level of chia oil in the diet considering their economic viability are necessary.

## Figures and Tables

**Table 1:** Ingredients and chemical composition (as-fed basis) of the pre- and experimental diets

Items	Pre-experimental diets		Experimental diets (29–42 days)			

	1–7 days	8–28 days	Soybean oil	Chia oil	Roasted whole soybean	Chia seed
Ingredients (g/kg)						
Corn	553.3	594.8	651.0	651.0	644.5	644.5
Soybean meal, 45%	381.8	340.6	292.5	292.5	160.0	160.0
Dicalcium phosphate	19.0	14.6	12.0	12.0	11.6	11.6
Limestone	9.1	9.1	8.0	8.0	7.7	7.7
Common salt	5.1	4.8	4.5	4.5	4.5	4.5
DL-methionine, 99%	3.65	2.7	2.5	2.5	2.5	2.5
L-lysine HCL, 99%	3.1	2.2	2.1	2.1	2.4	2.4
L -threonine	1.1	0.7	0.4	0.4	0.5	0.5
L -valine	0.0	0.0	0.2	0.2	0.4	0.4
Mineral supplement1)	0.5	0.5	0.5	0.5	0.5	0.5
Vitamin supplement2)	0.5	0.4	0.4	0.4	0.5	0.5
Choline chloride, 60%	0.5	0.45	0.4	0.4	0.4	0.4
Salinomycin, 12%	0.5	0.5	0.5	0.5	0.5	0.5
Soybean oil	21.8	28.6	25.0	0.0	0.0	0.0
Chia oil	0.0	0.0	0.0	25.0	0.0	0.0
Roasted whole soybean	0.0	0.0	0.0	0.0	164.0	0.0
Chia seed	0.0	0.0	0.0	0.0	0.0	164.0
Calculated3),5) and determined4),6) composition						
Apparent metabolizable energy3) (MJ/kg)	12.37	12.78	12.99	-	12.98	-
Apparent metabolizable energy4) (MJ/kg)	-	-	13.00	13.11	13.33	12.72
Crude protein (%)	21.703)	20.143)	18.753)	18.753)	18.733)	16.325)
Crude protein6) (%)	-	-	17.99	17.99	18.07	15.52
Digestible methionine + cysteine3) (%)	0.95	0.82	0.77	0.77	0.76	-
Digestible lysine3) (%)	1.33	1.16	1.04	1.04	1.04	-
Digestible threonine3) (%)	0.85	0.76	0.68	0.68	0.68	-
Digestible valine3) (%)	-	-	0.82	0.82	0.81	-
Total phosphorus	0.693)	0.603)	0.543)	0.543)	0.543)	0.615)
Available phosphorus (%)	0473)	0.383)	0.333)	0.333)	0.323)	0.345)
Calcium (%)	0.933)	0.813)	0.693)	0.693)	0.683)	0.725)
Sodium (%)	0.223)	0.203)	0.203)	0.203)	0.203)	0.195)
Acid detergent fiber6)	-	-	0.52	0.53	7.01	10.16
Neutral detergent fiber6)	-	-	1.71	1.70	19.20	23.81
Crude fiber6)	2.81	2.68	2.68	2.68	3.42	5.05
Feed price7) ($/kg)	-	-	0.31	0.70	0.32	0.51

1) Supplemented per kilogram of feed: Zn 55 mg; Se 0.18 mg; I 0.70 mg; Cu 10 mg; Mn 78 mg; Fe 48 mg.

2) Supplemented per kilogram of feed: folic acid 0.48 mg; pantothenic acid 8.70 mg; biotin 0.018 mg; butylhydroxytoluene (BHT) 1.5 mg; niacin 11.1; vitamin A 6,000 IU; vitamin B_1_ 0.8 mg; vitamin E 12.15 IU; vitamin B_12_ 8.10 μg; vitamin B_2_ 3.6 mg; vitamin B_6_ 1.80 mg; vitamin D_3_ 1,500 IU; vitamin K_3_ 1.44 mg.

3) Calculated according to Rostagno et al [11].

4) Determined in *in vivo* assay.

5) Calculated with values related by Jin et al [12].

6) Determined in laboratory assay.

7) Price per kg of ingredient: soybean oil 0.89$/kg, chia oil 16.39$/kg, roasted whole soybean 0.52$/kg, chia seed 1.72$/kg.

**Table 2:** Determined chemical composition and gross energy of the evaluated feedstuffs (as-fed basis)

Composition (%)	Soybean oil	Chia oil	Roasted whole soybean	Chia seed
Dry matter	99.85	100.0	92.96	92.78
Gross energy (MJ/kg)	39.49	39.23	22.30	24.53
Crude protein	-	-	32.32	17.83
Ether extract	-	-	18.53	27.04
Ash	-	-	4.24	3.64
Acid detergent fiber	-	-	15.55	34.75
Neutral detergent fiber	-	-	28.04	56.17
Crude fiber	-	-	8.81	30.80

**Table 3:** Fatty acid profile of soybean oil, chia oil, roasted whole soybean and chia seed

Fatty acids (%)	Soybean oil	Chia oil	Roasted whole soybean	Chia seed
C14:0	0.07	0.13	0.10	0.05
C15:0	0.09	0.10	0.03	0.04
C16:0	10.75	8.51	11.74	7.24
C16:1	0.12	0.39	0.19	0.30
C17:0	0.09	0.11	0.09	0.06
C17:1	0.04	0.05	0.06	0.02
C18:0	3.17	4.76	3.48	3.67
C18:1*n-9 cis*	25.07	10.58	26.13	7.42
C18:1*n-9 trans*	0.039	0.033	0.027	0.029
C18:2*n-6 cis*	53.48	17.78	50.33	19.76
C18:3*n-3*	5.55	56.33	6.44	60.40
C18:3*n-6*	0.11	0.24	0.03	0.23
C20:0	0.31	0.28	0.29	0.27
C20:1*n-9*	0.30	0.23	0.21	0.15
∑SFA	14.48	13.89	15.73	11.32
∑MUFA	25.57	11.28	26.61	7.91
∑PUFA	59.14	74.35	56.79	80.38
∑*ω-3*	5.55	56.33	6.44	60.39
∑*ω-6*	53.59	18.02	50.36	19.99
∑*ω-6*/∑*ω-3*	9.65	0.32	7.83	0.33

∑SFA, total saturated fatty acids; ∑MUFA, total monounsaturated fatty acids; ∑PUFA, total polyunsaturated fatty acids; ∑*ω-3*, total omega-3 fatty acids; ∑*ω-6*, total omega-6 fatty acids; ∑*ω-6*/∑*ω-3*, total omega-6 fatty acids/total omega-3 fatty acids ratio.

**Table 4:** Fatty acid profile of the experimental diets

Fatty acids (%)	Experimental diets			

	Soybean oil	Chia oil	Roasted whole soybean	Chia seed
C14:0	0.09	0.07	0.07	0.10
C15:0	0.04	0.15	0.04	0.06
C16:0	12.42	9.43	11.66	11.00
C16:1	0.28	0.15	0.21	0.38
C17:0	0.10	0.11	0.11	0.08
C17:1	0.08	0.06	0.02	0.05
C18:0	3.12	3.48	3.03	3.44
C18:1*n-9 cis*	28.72	16.48	27.95	21.24
C18:1*n-9 trans*	0.04	0.02	0.05	0.05
C18:2*n-6 cis*	50.02	28.40	47.94	34.32
C18:3*n-3*	3.61	37.81	4.87	26.97
C18:3*n-6*	0.06	0.15	0.02	0.11
C20:0	0.39	0.33	0.40	0.36
C20:1*n-9*	0.25	0.19	0.22	0.16
∑SFA	16.16	13.57	15.30	15.03
∑MUFA	29.40	16.89	28.45	21.88
∑PUFA	53.70	66.36	52.83	61.40
∑*ω-3*	3.61	37.81	4.87	26.97
∑*ω-6*	50.08	28.55	47.96	34.43
∑*ω-6*/∑*ω-3*	13.87	0.76	9.85	1.28

∑SFA, total saturated fatty acids; ∑MUFA, total monounsaturated fatty acids; ∑PUFA, total polyunsaturated fatty acids; ∑*ω-3*, total omega-3 fatty acids; ∑*ω-6*, total omega-6 fatty acids; ∑*ω-6*/∑*ω-3*, total omega-6 fatty acids/total omega-3 fatty acids ratio.

**Table 5:** Physicochemical parameters and lipid peroxidation of broiler breast and thigh

Variables	Experimental diets				SEM	p-value

	Soybean oil	Chia oil	Roasted whole soybean	Chia seed		
Breast						
pH 24 hours *postmortem*	5.93^a^	5.88^b^	5.79^c^	5.73^d^	0.02	<0.001
Luminosity (L*)	54.58	56.26	56.28	55.57	0.35	0.281
Red intensity (a*)	0.44^b^	0.38^b^	0.90^a^	0.69^a^	0.07	0.019
Yellow intensity (b*)	11.17^b^	12.50^a^	12.94^a^	12.97^a^	0.22	0.003
Color saturation index (C*)	11.18^b^	12.52^a^	12.98^a^	13.21^a^	0.24	0.002
Hue angle (h*)	87.73	88.03	86.13	86.95	0.29	0.080
Cooking loss (%)	27.82	29.50	27.37	27.16	0.35	0.062
Shear force (kgf)	1.38	1.51	1.67	1.60	0.07	0.557
Oxidation (malondialdehyde/kg)	0.20	0.18	0.20	0.18	0.02	0.936
Thigh						
pH 24 hours *postmortem*	6.34^b^	6.46^a^	6.46^a^	6.44^a^	0.01	0.001
Luminosity (L*)	52.42	52.37	52.15	53.30	0.32	0.622
Red intensity (a*)	3.69	3.94	4.65	3.33	0.19	0.064
Yellow intensity (b*)	12.14^b^	12.86^a^	13.28^a^	13.85^a^	0.22	0.034
Color saturation index (C*)	12.73	13.50	13.99	14.28	0.22	0.055
Hue angle (h*)	73.35^b^	73.86^b^	71.29^b^	76.71^a^	0.70	0.036
Cooking loss (%)	25.26^b^	24.57^b^	25.03^b^	28.94^a^	0.50	0.001
Shear force (kgf)	1.11	1.08	1.18	1.18	0.03	0.541
Oxidation (malondialdehyde/kg)	0.32^b^	0.51^a^	0.36^b^	0.37^b^	0.02	0.004

SEM, standard error of the mean.

a–d Means followed by different superscripted lowercase letters in the same row differ from each other (p<0.05) according to the Scott-Knott test.

**Table 6:** Proximate composition of broiler breast and thigh meat

Items	Experimental diets				SEM	p-value

	Soybean oil	Chia oil	Roasted whole soybean	Chia seed		
Breast						
Moisture	73.64	73.48	73.00	72.94	0.14	0.223
Protein	21.05^b^	21.26^b^	21.98^a^	22.10^a^	0.16	0.030
Lipid	2.25	2.43	2.32	2.15	0.04	0.113
Minerals	3.09^a^	2.88^b^	2.86^b^	2.81^b^	0.03	0.005
Thigh						
Moisture	71.51	70.97	71.17	71.29	0.12	0.446
Protein	19.74	19.65	19.75	19.39	0.06	0.138
Lipid	5.33	5.83	5.70	5.66	0.11	0.420
Minerals	3.48^b^	3.53^b^	3.40^b^	3.69^a^	0.03	0.009

SEM, standard error of the mean.

a,b Means followed by different letters in the row differ from each other (p<0.05) according to the Scott-Knott test.

**Table 7:** Fatty acid profile of the breast of broilers at 42 days of age

Fatty acids (%)	Experimental diets				SEM	p-value

	Soybean oil	Chia oil	Roasted whole soybean	Chia seed		
C6:0	0.044^a^	0.034^a^	0.044^a^	0.019^b^	0.003	0.001
C10:0	0.009	0.009	0.014	0.010	0.002	0.729
C12:0	0.035	0.030	0.031	0.030	0.001	0.166
C14:0	0.421	0.408	0.406	0.402	0.004	0.312
C14:1	0.095	0.097	0.099	0.0110	0.002	0.100
C15:0	0.396	0.381	0.348	0.226	0.025	0.055
C16:0	21.960^a^	20.947^b^	21.259^b^	21.796^a^	0.131	0.008
C16:1	3.522^b^	3.720^b^	3.454^b^	4.164^a^	0.081	<0.001
C17:0	0.249^a^	0.194^b^	0.217^b^	0.150^c^	0.010	<0.001
C17:1	0.253	0.206	0.225	0.1824	0.010	0.051
C18:0	8.438^a^	7.710^b^	7.825^b^	7.716^b^	0.111	0.046
C18:1*n-9 cis*	31.164	30.373	31.428	31.938	0.229	0.096
C18:1*n-9 trans*	0.179^a^	0.156^b^	0.155^b^	0.151^b^	0.003	0.002
C18:2*n-6 cis*	24.745^b^	21.505^c^	26.344^a^	20.262^d^	0.583	<0.001
C18:3*n-3*	2.006^c^	8.741^a^	2.087^c^	7.802^b^	1.81	<0.001
C18:3*n-6*	0.196^a^	0.165^b^	0.185^a^	0.163^b^	0.004	0.004
C20:0	0.076	0.085	0.080	0.073	0.002	0.194
C20:1*n-9*	0.322^a^	0.260^b^	0.294^a^	0.291^a^	0.008	0.023
C20:2	0.517^a^	0.358^b^	0.471^a^	0.371^b^	0.019	<0.001
C20:3*n-3*	0.061^b^	0.187^a^	0.052^b^	0.213^a^	0.017	<0.001
C20:3*n-6*	0.779^a^	0.570^b^	0.631^b^	0.607^b^	0.026	0.013
C20:4*n-6*	3.553^a^	2.236^b^	3.172^a^	2.555^b^	0.140	<0.001
C20:5*n-3*	0.171^c^	0.558^b^	0.141^c^	0.619^a^	0.051	<0.001
C22:0	0.095	0.087	0.079	0.096	0.004	0.474
C22:1*n-9*	0.030	0.022	0.022	0.026	0.001	0.108
C22:2	0.042^c^	0.098^b^	0.043^c^	0.116^a^	0.008	<0.001
C22:6*n-3*	0.326^b^	0.420^a^	0.289^b^	0.448^a^	0.019	<0.001
C24:0	0.021^a^	0.016^b^	0.014^b^	0.014^b^	0.001	<0.001
C24:1*n-9*	0.006	0.013	0.011	0.012	0.002	0.433
∑SFA	31.748^a^	29.903^b^	30.319^b^	30.532^b^	0.217	0.006
∑MUFA	35.571	34.847	35.688	36.874	0.273	0.053
∑PUFA	32.396^b^	34.837^a^	33.416^b^	33.156^b^	0.326	0.046
∑PUFA/∑SFA	1.023^b^	1.168^a^	1.106^a^	1.090^a^	0.017	0.011
∑*ω-3*	2.564^b^	9.905^a^	2.569^b^	9.082^a^	0.810	<0.001
∑*ω-6*	29.273^b^	24.475^c^	30.333^a^	23.586^c^	0.685	<0.001
∑*ω-6*/∑*ω-3*	11.620^a^	2.493^b^	11.879^a^	2.699^b^	1.055	<0.001
Δ9-desaturase^C16^	13.771^c^	15.042^b^	13.964^c^	16.011^a^	0.248	<0.001
Δ9-desaturase^C18^	72.166	71.669	72.342	72.658	0.147	0.105
Elongase^C16–C18^	60.860	60.692	61.368	60.446	0.152	0.174
Atherogenicity1)	0.369^a^	0.349^b^	0.359^b^	0.369^a^	0.003	0.034
Thrombogenicity2)	0.768^a^	0.488^c^	0.725^b^	0.524^c^	0.029	<0.001

SEM, standard error of the mean; ∑SFA, total saturated fatty acids; ∑MUFA, total monounsaturated fatty acids; ∑PUFA, total polyunsaturated fatty acids; ∑PUFA/∑SFA, total polyunsaturated fatty acids/total saturated fatty acids ratio; ∑*ω-3*, total omega-3 fatty acids; ∑*ω-6*, total omega-6 fatty acids; ∑*ω-6*/∑*ω-3*, total omega-6 fatty acids/total omega-3 fatty acids ratio.

1) Atherogenicity index.

2) Thrombogenicity index.

a–d Means followed by different letters in the row differ from each other (p<0.05) according to the Scott-Knott test.

**Table 8:** Fatty acid profile of the thigh of broilers at 42 days of age

Fatty acids (%)	Experimental diets				SEM	p-value

	Soybean oil	Chia oil	Roasted whole soybean	Chia seed		
C6:0	0.038	0.035	0.036	0.036	0.002	0.942
C10:0	0.016	0.010	0.006	0.008	0.002	0.587
C12:0	0.029	0.034	0.028	0.031	0.001	0.068
C14:0	0.405	0.392	0.402	0.398	0.005	0.863
C14:1	0.105	0.096	0.106	0.107	0.002	0.427
C15:0	0.342	0.279	0.265	0.288	0.012	0.081
C16:0	20.819	20.234	20.537	20.791	0.110	0.210
C16:1	4.186	3.985	4.369	4.434	0.082	0.214
C17:0	0.181^a^	0.188^a^	0.159^b^	0.190^a^	0.004	0.027
C17:1	0.199	0.199	0.149	0.179	0.009	0.192
C18:0	7.753	8.115	3.380	7.892	0.126	0.221
C18:1*n-9 cis*	31.633^a^	29.892^b^	31.662^a^	30.281^b^	0.242	0.004
C18:1*n-9 trans*	0.161	0.154	0.144	0.165	0.003	0.052
C18:2*n-6 cis*	26.959^a^	23.427^b^	26.781^a^	21.694^c^	0.540	<0.001
C18:3*n-3*	1.850^b^	7.314^a^	1.999^b^	6.873^a^	0.600	<0.001
C18:3*n-6*	0.196^a^	0.157^b^	0.190^a^	0.173^b^	0.005	0.004
C20:0	0.076^b^	0.099^a^	0.082^b^	0.079^b^	0.003	0.006
C20:1*n-9*	0.272	0.275	0.278	0.284	0.004	0.749
C20:2	0.356	0.331	0.358	0.366	0.006	0.214
C20:3*n-3*	0.050^c^	0.173^a^	0.045^c^	0.150^b^	0.013	<0.001
C20:3*n-6*	0.579	0.572	0.531	0.546	0.010	0.304
C20:4*n-6*	3.419	2.991	3.172	2.999	0.086	0.267
C20:5*n-3*	0.097^b^	0.557^a^	0.112^b^	0.537^a^	0.052	<0.001
C22:0	0.122	0.106	0.112	0.119	0.003	0.293
C22:1*n-9*	0.019	0.018	0.018	0.020	0.0005	0.449
C22:2	0.034^b^	0.104^a^	0.042^b^	0.100^a^	0.008	<0.001
C22:6*n-3*	0.245^c^	0.464^a^	0.244^c^	0.368^b^	0.023	<0.001
C24:0	0.010	0.012	0.010	0.009	0.001	0.813
C24:1*n-9*	0.007	0.011	0.009	0.008	0.001	0.783
∑SFA	29.791	29.505	29.017	29.843	0.175	0.340
∑MUFA	36.581^a^	34.629^b^	36.733^a^	35.478^b^	0.278	0.010
∑PUFA	33.787^b^	36.091^a^	33.477^b^	33.804^b^	0.310	0.002
∑PUFA/∑SFA	1.137^b^	1.226^a^	1.155^b^	1.136^b^	0.013	0.045
∑*ω-3*	2.243^b^	8.507^a^	2.402^b^	7.927^a^	0.686	<0.001
∑*ω-6*	31.154^a^	27.148^b^	30.675^a^	25.412^c^	0.569	<0.001
∑*ω-6*/∑*ω-3*	14.110^a^	3.207^c^	12.840^b^	3.286^c^	1.185	<0.001
Δ9-desaturase^C16^	16.656	16.421	17.520	17.544	0.240	0.226
Δ9-desaturase^C18^	72.473^a^	71.340^b^	72.485^a^	71.581^b^	0.159	0.004
Elongase^C16–C18^	61.187	61.088	61.054	60.210	0.193	0.258
Atherogenicity1)	0.353^a^	0.333^b^	0.355^a^	0.352^a^	0.003	0.020
Thrombogenicity2)	0.713^a^	0.508^b^	0.692^a^	0.537^b^	0.022	<0.001

SEM, standard error of the mean; ∑SFA, total saturated fatty acids; ∑MUFA, total monounsaturated fatty acids; ∑PUFA, total polyunsaturated fatty acids; ∑PUFA/∑SFA, total polyunsaturated fatty acids/total saturated fatty acids ratio; ∑*ω-3*, total omega-3 fatty acids; ∑*ω-6*, total omega-6 fatty acids; ∑*ω-6*/∑*ω-3*, total omega-6 fatty acids/total omega-3 fatty acids ratio.

1) Atherogenicity index.

2) Thrombogenicity index.

a–c Means followed by different letters in the row differ from each other (p<0.05) according to the Scott-Knott test.

**Table 9:** Performance of broilers fed diets containing soybean (oil or grain) or chia (oil or seeds) from 29 to 42 days of age

Items	Experimental diets				SEM	p-value

	Soybean oil	Chia oil	Roasted whole soybean	Chia seed		
FI (kg/broiler)	2.905^a^	2.636^b^	2.548^b^	2.984^a^	0.016	<0.001
WG (kg/broiler)	1.518^a^	1.477^a^	1.399^b^	1.383^b^	0.045	<0.001
FCR (kg/kg)	1.91^b^	1.78^a^	1.82^a^	2.14^c^	0.034	<0.001

SEM, standard error of the mean; FI, feed intake; WG, weight gain; FCR, Feed conversion ratio.

a–c Means followed by different letters in the row differ from each other (p<0.05) according to the Scott-Knott test.
